# Impact of an antibiotic stewardship program on antibiotic utilization, bacterial susceptibilities, and cost of antibiotics

**DOI:** 10.1038/s41598-023-32329-6

**Published:** 2023-03-28

**Authors:** Banan M. Aiesh, Maisa A. Nazzal, Aroub I. Abdelhaq, Shatha A. Abutaha, Sa’ed H. Zyoud, Ali Sabateen

**Affiliations:** 1 Infectious Disease Unit, An-Najah National University Hospital, Nablus, 44839 Palestine; 2 Department of Clinical and Community Pharmacy, College of Medicine and Health Sciences, An-Najah National University, Nablus, 44839 Palestine; 3 Department of Medicine, College of Medicine and Health Sciences, An-Najah National University, Nablus, 44839 Palestine; 4 Clinical Research Center, An-Najah National University Hospital, Nablus, 44839 Palestine

**Keywords:** Microbiology, Diseases, Health care

## Abstract

Antimicrobial misuse is a worldwide issue, and antimicrobial resistance is considered the most challenging aspect of health care. It has been reported that as much as 30–50% of antimicrobials prescribed in hospitals are deemed unnecessary or inappropriate. Antibiotic stewardship programs (ASPs) include policies that apply continuous management of judicious anti-infectious treatment in the clinical setting. Therefore, the objectives of this study were to evaluate the effect of ASPs on antibiotic consumption, the costs of antibiotic expenditure, and the sensitivity of antimicrobials. A retrospective, quasi-experimental study was performed to assess the effect of ASP at An-Najah National University Hospital, a tertiary care hospital in the West Bank, Palestine, over a period of 20 months before and 17 months after the implementation of the ASP. Data on antibiotic consumption were reported monthly as days of therapy per 1000 patient-days and monthly costs (USD/1000 patient-days). A total of 2367 patients who received one or more of the targeted antibiotics (meropenem, colistin and tigecycline) during their hospital stay were included in the study. They have split into two groups: 1710 patients in the pre-ASP group, and 657 patients in the post ASP group. The most significant reduction in DOT per 1000 patient-days was seen with tigecycline, with a percentage of change of − 62.08%. Furthermore, the mean cost of the three antibiotics decreased significantly by 55.5% in the post-ASP phase compared to the pre-ASP phase. After the implementation of ASP, there was a statistically significant increase in susceptibility to meropenem, piperacillin and piperacillin/tazobactam with respect to *Pseudomonas aeruginosa*. However, changes in mortality rates were not statistically significant (*p* = 0.057). ASP positively reduced costs and antimicrobial consumption, with no statistically significant effect on the overall mortality rate. However, a long-term evaluation of the ASP's impact is needed to conclude its lasting impact on infection-related mortality and antimicrobial susceptibility pattern.

## Introduction

Misuse and overuse of antimicrobial agents are dangerous phenomena, and increases in antibiotic resistance among hospital-acquired pathogens are driven mainly by increased antibiotic utilization in the hospital setting^1^. Moreover, that was strongly associated with an increased risk of the emergence and spread of multidrug-resistant organisms (MDRO) to others within the hospital or the community. As a result, the role of antibiotic stewardship programs (ASPs) has arisen, and implementing such programs has led to better outcomes.

As reported, nearly 30–50% of hospital antibiotics are considered unnecessary or inappropriate^2^. Studies conducted in the 1970s showed that antimicrobials were not used appropriately in acute care settings. Around 14–43% of all prescribed antimicrobial therapy courses were not considered necessary, since there was no apparent source of infection^2^. This transom persisted over the decade, and in US acute care hospitals, about 30% of prescribed antimicrobials are either not necessary or suboptimal^2^. Moreover, this inappropriate use increases antibiotic pressure and shortages. Meanwhile, few new antimicrobial agents are available, meaning clinicians are left with scanty effective therapeutic options for their patients^3^. This is also correlated with an increased risk of toxicities and adverse drug effects.

In Palestine, we lack studies investigating the impact of ASPs on antimicrobial utilization and the emergence of antimicrobial resistance (AMR). Therefore, our study was the first to assess the influence of antimicrobial stewardship programs on antibiotic consumption, cost, and antimicrobial resistance. ASP was implemented in September 2019 based on the Infectious Diseases Society of America (IDSA) and the Society for Healthcare Epidemiology of America (SHEA) guidelines, in an attempt to reduce the use of restricted antimicrobials. After establishing our ASP, 4 Ds of optimal antimicrobial therapy were implemented and followed: right Drug, right Dose, De-escalation to pathogen-directed therapy and right Duration of therapy. In addition, our implementation took into account the significant variations in medical practice, the repercussions on clinical outcomes and mortality rates among patients, multi-drug resistance of major pathogens, and utilization of antibiotics pre-and post- ASP implementation.

Our objectives were to evaluate the effect of ASPs on antibiotic consumption, antibiotic expenditure costs, and antimicrobial sensitivity patterns. In addition, we aimed to enhance patient outcomes and safety by using the most suitable antimicrobials, identifying the economic impact of ASPs, and assessing the effects of ASPs on the in-hospital mortality rate.

## Methods

### Study design

A retrospective and quasi-experimental study, introduced in September 2019, was performed to determine the effect of our ASP on three restricted antimicrobials’ (meropenem, colistin, and tigecycline) consumption, costs, and the hospital’s antibiogram. We compared the consumption, cost, and antibiogram pattern over a period that encompassed 20 months before (1 January, 2018–31 August, 2019) and 16 months after the implementation of the ASP (1 September, 2019–1 December, 2020).

In our hospital, antibiotic stewardship was delivered by in-person coaching by an infectious disease (ID) clinical pharmacist and an ID specialist. The ID specialist made two rounds per week with the ID clinical pharmacist. The ID clinical pharmacist with direct contact with ID specialist performed the remaining weekly rounds. The clinical rounds were held six days per week. Consultations were available by calling the ID team on holidays and off hours. The pre-authorization process was implemented as policy in the hospital setting by which the list of restricted antibiotics was stated in addition to the approval process agents. The order approval and sign by the ID team is done within 24 h. Exceptions for giving the initial or STAT doses were clarified in the policy as in septic shock cases for patients with a history of microorganisms that necessitate using any of the targeted three agents.

### Study setting

The study was conducted at An-Najah National University Hospital, a 120 bedded tertiary care hospital that provides surgical, medical, oncological and hemodialysis services for adults and pediatrics, and is considered one of the Palestinian’s leading institutions in the field of health care, in the West Bank, Palestine. So, an institutionally supported educational ASP was implemented in An-Najah National University Hospital, a non-profit tertiary care center hospital and the only teaching hospital in Palestine with 120 beds that provides several medical services. More than 60% of the services are provided for oncology patients—both pediatrics and adults—in the forms of departments, intensive care units (ICUs) and bone marrow transplant (BMT) units. The remaining services are divided between the surgical ward, surgical ICU, and cardiology units^4^.

### Study population and sample size

All patients admitted to the hospital who received at least one of the three restricted antibiotics between January 1 2018 and December 31 2020 from all hospital departments were included in this study.

### Data collection instrument

Information about patients who received antimicrobials was obtained from the hospital’s patient information system and pharmacy computer system. In addition, data regarding antibiotic consumption was collected using an excel sheet and reported as days of therapy per 1000 patient-days, together with monthly costs (USD/1000 patient-days). All patients receiving antibiotics had their history and course of the disease, laboratory and microbiological findings, indications, and drug choices evaluated during the rounds. Daily direct contact with the microbiology team was obtained to detect preliminary and final microbiologic culture results, thus guiding the selection of definitive therapy. Regarding the cause of selecting these three agents to work on is based on available agents in the formulary list, the cost, and the broadness of the spectrum of activity of the agents. Actually, these agents are considered the last option for treating resistant pathogens in our developing country. The other agents approved for MDR pathogens management were unavailable in the setting, and these three agents were considered our major resources. For colistin, it is the only available agent for carbapenem-resistant Enterobacterales (CRE) or MDR *Pseudomonas aeruginosa* treatment, for example. Same for Vancomycin-resistant *Enterococcus* (VRE) management, only tigecycline and linezolid (that is not always available due to very high cost compared with tigecycline despite its superiority to tigecycline). So we aimed to control the prescription of these agents that are considered our last reservoir as the new or approved other agents are not available in Palestine.

### Statistical analysis

The Statistical Package for Social Sciences program (IBM-SPSS), version 21, was used to enter and analyze patient data. Testing for data normality was done via the Kolmogorov–Smirnov test. Student t-test (independent sample t-test) was used for normally distributed data, while the Mann–Whitney U test (two independent sample test) was used for non-normally distributed data. In addition, a Chi-square test was used to determine the relationship between antimicrobial susceptibility and the year the microbe was isolated to determine the trend of susceptibility over time. For all tests, *p*-values < 0.05 were considered significant.

### Ethics approval and consent to participate

The *Institutional Review Boards (IRB) of An-Najah National University* had authorized all components of the study protocol, including access to and use of patient clinical data*.* We can confirm that the information gathered was only used for clinical research. The information was kept private and was only utilized for the purposes of this study. The data was acquired with only limited access to the project's working staff. We did not share identifiable patient information; we used numbers as codes for patients instead of their names. All experiments in our study were performed in accordance with relevant guidelines and regulations. Because we used retrospective data, *IRB of An-Najah National University* waived the requirement for informed consent.

## Results

This study included a total of 2367 patients who took at least one of the targeted antibiotics (meropenem, colistin, and tigecycline) during their hospital stay. These included patients admitted to the hospital's different departments. They were divided into two groups: 1710 patients in the pre-ASP group and 657 patients in the post-ASP group. In addition, they received at least one of the restricted antibiotics.

For the selected antimicrobials, there was a statistically significant difference in days of therapy (DOT) per 1000 patient-days between the two groups. The most significant reduction in DOT per 1000 patient-days was seen with tigecycline, with a percentage of change of − 62.08%, as shown in Table 1. Furthermore, the percentage of change was − 59.46% for colistin and − 38.03% for meropenem.

**Table 1:** Days of therapy (DOT) per 1000 patient-days for the selected antibiotics during the two phases of the study (pre- and post-intervention).

Antimicrobial agent	Mean ± SD/median (IQR) of DOT per 1000 patient-days		*p*-value	Percent of change
	Pre-ASP	Post-ASP		
Colistin	86.97 ± 22.46	35.26 ± 13.81	< 0.001^a^	− 59.46%
Meropenem	142.01 (105.84–157.60)	88.00 (72.63–107.00)	0.018^b^	− 38.03%
Tigecycline	45.33 ± 11.97	17.19 ± 9.37	< 0.001^a^	− 62.08%

*DOT* days of therapy, *ASP* antibiotic stewardship program, *SD* standard deviation.

^a^Analyzed using independent sample t-test.

^b^Analyzed using the Mann–Whitney U test.

The mean cost of the three antibiotics dropped significantly by 55.5% in the post-ASP phase, as shown in Table 2. Furthermore, an average of 5669.21 USD was saved during the intervention phase. The highest cost saved was with regards to tigecycline consumption, showing a reduction of 62.07% in mean hospital cost attributed to its use.

**Table 2:** Comparison of the mean/median costs of antimicrobial administration in the average value of Israeli Shekel during the two phases of the study (pre- and post-intervention).

Antimicrobial agent	Mean ± SD/median (IQR) of cost per 1000 patients-day		*p*-value	Percent of change
	Pre-ASP	Post-ASP		
Colistin	2079.62 ± 536.98	843.12 ± 330.26	< 0.001^a^	− 59.46%
Meropenem	2430.63 (1840.78–2740.87)	1539.13 (1265.22–1928.26)	0.026^b^	− 36.68%
Tigecycline	5715.24 ± 1509.47	2168.03 ± 1181.92	< 0.001^a^	− 62.07%
Total	10,225.49	4556.28		− 55.5%

*ASP* antibiotic stewardship program, *SD* standard deviation, *IQR* interquartile range.

^a^Analyzed using independent sample t-test.

^b^Analyzed using the Mann–Whitney U test.

The mortality rate was evaluated in all patients in the hospital during the study period, spanning from January 2018 to December 2020. The total mortality rate fluctuated throughout that period; the mortality mean was 2.34 ± 0.57 in the pre-ASP period, while the mean was 2.88 ± 0.95 in the post-ASP period. However, these changes in mortality rates were not statistically significant (*p* = 0.057).

Trends in antibiotic sensitivity were observed between January 2018 and December 2020. Regarding *Pseudomonas aeruginosa*, a statistically significant increase was found regarding susceptibility to amikacin, gentamicin, tobramycin, ciprofloxacin, meropenem, piperacillin and piperacillin/tazobactam (*p* < 0.05). At the same time, there was no significant change in susceptibility to ceftazidime. Meanwhile, there was a statistically significant decrease in imipenem susceptibility (*p* < 0.001); (Table 3).

**Table 3:** Antibiotic Sensitivity Trends from 2018 to 2020.

Microorganism	Antimicrobial agent	2018			2019			2020			P value*
		%	S	R	%	S	R	%	S	R	
*P. aeruginosa*	Ceftazidime	66	99	51	66.2	110	56	75	87	29	0.213
	Amikacin	67.7	103	49	69	114	51	84.6	99	18	***0.003***
	Gentamycin	63.8	97	55	62.4	103	62	76.9	90	27	***0.024***
	Tobramycin	65.7	96	50	70	112	48	82.7	91	19	***0.009***
	Ciprofloxacin	57.6	87	64	56.9	94	71	72.6	85	32	***0.014***
	Meropenem	60.5	92	60	60.6	100	65	80.3	94	23	***0.001***
	Imipenem	60.2	91	60	36.3	60	105	54.7	64	53	** < 0.001**
	Piperacillin/Tazobactam	72.9	97	36	75.4	120	39	88.5	101	13	***0.006***
	Piperacillin	47.5	70	76	56.9	91	69	81.4	88	20	** < *0.001***
*K. pneumoniae*	Meropenem	86	117	19	76.4	136	42	74.4	134	46	**0.034**
	Amikacin	86.6	117	18	76.2	135	42	77.7	140	40	**0.022**
	Gentamycin	56.6	77	59	54.4	97	81	59.4	107	73	0.638
	Ciprofloxacin	50.3	68	67	44.3	79	99	44.4	80	98	0.524
	Ceftriaxone	38.5	52	83	33.7	60	118	30	54	126	0.286
	Trimethoprim-sulfamethoxazole	41.4	56	79	35	55	122	30.8	55	123	0.090
	Nitrofurantoin	16.3	8	41	13.7	8	50	16	9	47	0.920
	Piperacillin/Tazobactam	61.4	83	52	56.4	102	72	56.6	102	78	0.691
	Amoxicillin/clavulanic acid	40	54	81	39.3	70	108	35.5	64	116	0.666
*S. aureus*	Oxacillin	50.5	49	48	49.1	57	59	56.1	55	43	0.569
	Vancomycin	100	99	0	99.2	125	1	100	98	0	0.456
	Tigecycline	100	99	0	100	126	0	100	98	0	-
	Linezolid	100	99	0	100	126	0	100	98	0	-
	Rifampicin	98.9	96	1	97.6	123	3	98.9	97	1	0.632
	Tetracycline	81.8	81	18	79.2	99	26	89.6	87	10	0.106
	Clindamycin	76.7	76	23	74.1	92	32	76	73	23	0.898
	Erythromycin	63.6	63	36	54.8	68	56	64.5	55	41	0.404
	Ciprofloxacin	71.7	71	28	65.6	82	43	77.3	75	22	0.159
	Levofloxacin	75.7	75	24	65.6	82	43	76.2	74	23	0.128
	Moxifloxacin	78.7	78	21	84.8	106	19	96.9	94	3	***0.001***
	Gentamicin	92.9	92	7	92	116	10	95.9	94	4	0.491
	Trimethoprim-sulfamethoxazole	90.3	84	9	89.3	109	13	93.8	92	6	0.482
	Quinopristin/dalfopristin	98.9	98	1	100	126	0	100	98	0	0.321
*E. coli*	Amoxicillin/clavulanic acid	44.5	114	142	48.8	146	153	44.7	131	162	0.502
	Meropenem	98.4	253	4	98.3	292	5	94.8	277	15	**0.014**
	Amikacin	84.2	219	41	93.9	281	18	97.5	287	6	** < *0.001***
	Gentamycin	70	182	78	63.5	190	109	71.2	208	84	0.100
	Ceftriaxone	41.5	108	152	46.4	138	159	45.3	133	160	0.477
	Piperacillin/Tazobactam	80.6	208	50	80.9	242	57	77.3	226	66	0.505
	Ciprofloxacin	39.2	102	158	37.4	112	187	41	120	172	0.664
	Nitrofurantoin	88.8	127	16	90.7	138	13	93.1	135	10	0.436
	Trimethoprim-sulfamethoxazole	43.7	112	144	35.4	106	193	53.4	102	189	0.064
*E. faecium*	Ciprofloxacin	7.8	9	106	4.8	5	99	4.2	5	113	0.448
	Levofloxacin	9.5	11	104	6.6	7	98	5.9	7	111	0.538
	Tigecycline	98.2	113	2	99	104	1	99.1	116	1	0.795
	Tetracycline	60	69	46	59	62	43	58.9	69	48	0.985
	Vancomycin	46	53	62	36.1	38	67	38.9	46	72	0.300
	Linezolid	97.2	107	3	97	100	3	97.4	115	3	0.986
	Quinopristin/dalfopristin	85.2	98	17	80	84	21	5	105	13	0.173
*E. faecalis*	Ampicillin	98.6	149	2	97.6	165	4	99.2	140	1	0.480
	Ciprofloxacin	43.9	69	88	40.8	69	100	44.6	63	78	0.761
	Levofloxacin	47.1	74	83	43.1	73	96	46	65	76	0.760
	Tigecycline	100	151	0	100	166	0	100	140	0	-
	Tetracycline	14.7	23	133	10.7	18	151	13.6	19	120	0.521
	Vancomycin	99.3	156	1	100	169	0	99.2	140	1	0.563
	Linezolid	97.3	146	4	95.1	156	8	94.8	128	7	0.502
	Nitrofurantoin	93.3	14	1	98.3	59	1	95	57	3	0.509
*Proteus spp*	Ceftriaxone	75.6	28	9	81.3	47	11	84.3	27	5	0.652
	Amoxicillin/clavulanic acid	82.7	24	5	77.7	42	12	72.4	21	8	0.639
	Piperacillin/Tazobactam	86.1	31	6	98.3	58	1	96.8	31	1	***0.012***
	Trimethoprim-sulfamethoxazole	24.3	9	28	37.2	22	37	37.5	12	20	0.367
	Ertapenem	96.8	31	1	100	56	0	96.8	31	1	0.411
	Meropenem	89.1	33	4	96.6	57	2	96.8	31	1	0.237
	Amikacin	100	37	0	98.3	58	1	100	32	0	0.555
	Gentamycin	64.8	24	13	52.2	31	28	71.8	23	9	0.166
	Ciprofloxacin	54	20	17	49.1	29	30	56.2	18	14	0.787
*A. baumannii*	Imipenem	9.3	12	117	17	15	73	8.9	7	71	0.153
	Meropenem	9.3	12	117	17	15	73	8.9	7	71	0.153
	Cefepime	8.5	11	118	13.6	12	76	10.2	8	70	0.482
	Piperacillin/Tazobactam	7.1	9	118	15.9	14	74	10.2	8	70	***0.042***
	Gentamycin	12.4	16	113	21.5	19	69	15.3	12	66	0.190
	Tobramycin	12.7	16	110	23.2	20	66	24.3	19	59	0.057
	Ciprofloxacin	8.5	11	118	13.6	12	76	7.6	6	72	0.352
	Minocycline	54.5	66	55	58.6	51	36	65.3	51	27	0.317
	Trimethoprim-sulfamethoxazole	39.5	49	75	37.8	13	51	28.7	21	52	**0.022**

S, number of susceptible pathogens; R, number of resistant pathogens; %, susceptibility rate; –, not applicable.

*Chi-square test; bold denotes decreasing susceptibility; bold italic denotes increasing susceptibility.

A statistically significant decrease in carbapenem susceptibility was observed for both *Klebsiella pneumoniae* (*p* = 0.034) and *Escherichia coli* *(p* = 0.014). However, while *K. pneumoniae* had a significant decrease in susceptibility to amikacin (*p* = 0.022), *E. coli* exhibited an increased susceptibility to amikacin (*p* < 0.001). As for other gram-negative bacterial isolates, there was a significant increase in the susceptibility of both *Proteus* spp. and *Acinetobacter baumannii* to piperacillin/tazobactam (*p* = 0.012, and 0.042, respectively). Meanwhile, *A. baumannii* significantly decreased susceptibility to trimethoprim-sulfamethoxazole (*p* = 0.022) (Table 3).

In terms of Gram-positive isolates, *Staphylococcus aureus* exhibited a significant increase in susceptibility to moxifloxacin (*p* = 0.001) while *Enterococcus faecalis* and *Enterococcus faecium* did not have a significant change in its susceptibility to antibiotics during the study period (Table 3).

## Discussion

Antibiotics have revolutionized the practice of medicine. They have transformed once-lethal infections into treatable illnesses and paved the way for other treatments, allowing chemotherapy, bone marrow transplants, and other medical advances to become possible. However, the misuse and abuse of antibiotics in inpatient and outpatient settings have created the growing problem of antibiotic resistance. The emergence of MDR organisms has cast an ominous shadow over hospitals worldwide, posing a new threat to patients. Hence, ASPs have become essential in successfully treating infections, safeguarding patients from the risks of needless antibiotic usage, and improving clinical outcomes while lowering hospital expenses and length of stay^5^.

The approaches include front-end or pre-prescription review of restricted antimicrobials that require prior authorization for use^6^. Furthermore, depending on available microbiology data and clinical aspects of the patient, prospective back-end reviews of existing antibiotic regimens were used to supply clinicians with recommendations to continue, adjust, change, or terminate medication^6^. An important aspect of the program was active learning sessions and lectures for resident physicians and other healthcare workers, in addition to patient education^7^. Policies and guidelines of clinical practice were implemented. The program's major purpose was to reduce the use of empiric carbapenems and increase the usage of narrower spectrum beta-lactamases, by de-escalating broad-spectrum empiric therapy early based on culture results, in what are called carbapenem-preserving regimens.

Prior to implementing our ASP, reviews of monthly dispensing reports for tigecycline, colistin, and meropenem revealed that frequent empiric use and prolonged durations of therapy of these agents were concerns for our hospital. At our hospital, which mainly serves oncology patients, a study was conducted on patients with hematological malignancies to determine the microbial profile of infections. The study showed that 34.8% of the isolated Gram-negative pathogens were extended-spectrum beta-lactamase-producing organisms, and 31.8% were CRE^8^. Another study was also performed on patients with solid organ malignancy that showed approximately 52.4% of the isolates were extended-spectrum beta-lactamase-producing *E. coli*. About 83.3% of *Pseudomonas aeruginosa* pathogens were sensitive to piperacillin-tazobactam and gentamicin, while 50% of the isolated *Pseudomonas aeruginosa* was resistant to imipenem^9^.

Bacterial resistance to antimicrobials is an emerging problem worldwide due to ineffective durations of therapy and inappropriate antimicrobial choices. It is a challenge that is associated with high morbidity and mortality rates. Furthermore, MDR bacteria are causing infections to become untreatable with conventional antibiotics^10,11^. As a result, antimicrobial resistance is expected to cause 10 million deaths annually by 2050, making it one of the main causes of mortality, with an economic burden of up to 100 trillion dollars^12^. From there comes the role of ASP, as they can optimize the clinical outcomes of patients by directing attention to the etiologic pathogen and its susceptibility profile while providing the appropriate drug of choice^13^. Furthermore, several studies have shown that ASP has many beneficial impacts on the rational use of antibiotics and reducing costs in healthcare settings^14–17^.

Regarding antibiotic consumption and the effects of ASP, our results showed that days of therapy per 1000 patient-days were reduced for the selected antimicrobials after program implementation. The most significant reduction was observed with tigecycline, with a percentage of change of − 62.08%. This impact of ASP on the utilization of antimicrobials was found in 24 studies with a rate of 11–38%^18^. Furthermore, meropenem was the antibiotic most frequently used prior to the application of the ASP, with a median DOT/1000 patient-days of 142.01 (105.84–157.60) before ASP. After ASP implementation, the median was reduced to 88 (72.63–107), reducing meropenem use by 38%. Similar results were seen in another study, in which meropenem was the second most commonly used antibiotic, and its usage was reduced by 50% after the implementation of the program^18^. Another study conducted in Spain showed a similar reduction in meropenem consumption^19^. ASP has many benefits beyond the reduction in antimicrobial utilization. It can indirectly help reduce hospital stays, accelerate the transition from intravenous to oral antimicrobials, reduce the risk of MDR organisms, reduce the incidence of adverse effects, and discontinue unnecessary antimicrobial agents.

As ASPs are designed to optimize patients’ clinical outcomes, multiple factors should be considered when selecting the antimicrobial agent, such as demographic characteristics, the severity of the patient’s illness, provision for the healthcare provider, and the level of adherence to the ASP guidelines^20^.

The program's benefits were observed not only in the clinical aspect but also in the financial aspect, where the mean cost of selected antimicrobials was significantly reduced by 55.5% in the post-ASP phase. Furthermore, the average cost saved during the intervention phase was 5669.21 USD. These results are similar to those seen in another study, in which the cost reduction in cost was 41.3%^21^. The impact of ASP in our hospital throughout the study period was significant in terms of reducing the consumption of the mentioned antibiotics and the related costs. Although there was a slight increase in overall mortality in the post-ASP period, the change in the mortality rate was not statistically significant, in agreement with results found in other studies^22,23^. The increase in overall mortality rate after September 2019 could be attributed to the pandemic of Coronavirus disease 2019 (COVID-19) as the hospital provided services for COVID-19 patients through a dedicated department and ICU and as the study that was performed to study the impact of COVID-19 on healthcare care-associated infections in intensive care units in low- and middle- income countries showed mortality rates were 15.2% and 23.2% for 2019 and 2020 (p < 0.0001), respectively^24^.

Cumulative antibiogram reports are necessary to monitor rising trends in resistance and to influence clinical decisions and infection control interventions^25^. Trends in antibiotic sensitivity were observed during the study period, between January 2018 and December 2020. Regarding *P. aeruginosa*, there was a significant increase in susceptibility to all antibiotics tested except ceftazidime, which showed no change, and imipenem, where a decrease in susceptibility was observed throughout the study period. A study conducted in Qatar showed that susceptibility to certain antimicrobials improved after the ASP was implemented in August 2015. The prevalence of MDR *P. aeruginosa* showed a sustained decrease from 2014 (9%) to 2017 (5.46%) (*p* = 0.019). There was a 23.9% reduction in studied antimicrobial consumption following ASP implementation (*p* = 0.008)^26^. In contrast to our findings in terms of *P. aeruginosa* sensitivity, the resistance of *P. aeruginosa* to imipenem and meropenem dropped significantly from 76.0 to 38.5% (*p* = 0.019) and from 74.1 to 30.0% (*p* = 0.012), respectively in the study done by Abdallah et al., 2017. The susceptibility pattern of *P. aeruginosa* to other antibacterial was not affected by carbapenem restriction^27^.

Our study also found a significant decrease in the susceptibility of *E. coli* and *K. pneumoniae* to carbapenems. This is in contrast to the Greek study in which colistin-resistant *K. pneumoniae* decreased from 31.9% in 2015 to 22.1% in 2016 (*p* = 0.10) and colistin-resistant *A. baumannii* from 13.8% in 2015 to 8.1% in 2016. Additionally, while *K. pneumoniae* isolates showed decreasing amikacin susceptibility, *E. coli* isolates showed increasing amikacin susceptibility. The evidence suggests that the decrease of *E. coli* and *K. pneumoniae* sensitivity to meropenem is due to the prevalence of carbapenem-resistant *Enterobacteriaceae* in our hospital. As a referral hospital, we deal with a large number of patients who have been infected or colonized with these resistant bacteria, resulting in secondary infections. This high burden of carbapenem-resistant *Enterobacteriaceae* is likely to have contributed to the decline in meropenem sensitivity of these two bacterial species^28,29^. As a result, we must take this issue seriously and implement appropriate solutions, such as antibiotic stewardship programs and infection control practices. We can reduce the spread of resistant bacteria and improve the efficacy of antibiotic treatments for all patients by doing so. The COVID-19 pandemic has undoubtedly presented unprecedented challenges to the global healthcare system^30^. One of the most concerning consequences of the pandemic has been an increase in antibiotic consumption^31,32^, both in the community and in other hospitals. This rise in antibiotic use could have a significant impact on the prevalence of MDR isolates in our hospital. As a result, it is critical that we remain vigilant and take all necessary steps to reduce the overuse of antibiotics, which could exacerbate the already dire situation of antibiotic resistance. Regarding *A. baumannii*, our study found an increase in susceptibility to piperacillin/tazobactam and a decrease in susceptibility to trimethoprim-sulfamethoxazole, while in the study performed by Chamieh et al., 2019 the overall extensively drug-resistant*A. baumanii* isolation decreased by 64.7% from period pre to post ASP periods. In addition, isolates from post-ASP period were more antimicrobial-susceptible: 64.8% sensitive to ceftazidime and cefepime, 17.6% to piperacillin/tazobactam, and 17.6% to carbapenems^33^.

Regarding Gram-positive bacteria, our study found a significant increase in *S. aureus* susceptibility to moxifloxacin and a non-significant decrease in the susceptibility of *E. faecium* to vancomycin. Meanwhile, a Greek tertiary care hospital study showed a reduction of vancomycin-resistant enterococci (VRE) from 22.5% in 2015 to 18.2% in 2016 (*p* = 0.52)^34^.

### Limitations

Our study has some limitations, including the lack of demographic data of the patients and our study is limited to a quasi-experimental pre-and post-implementation design and is limited to a single center. In addition, there wasn’t a washout period while the ASP was getting up and running in the first months. Another limitation was the non-equality of the months between the pre- and post-intervention periods.

## Conclusions

This study demonstrated that the ASP positively reduced costs and antimicrobial consumption, with no statistically significant effect on the mortality rate. However, a long-term evaluation of the ASP's impact is needed to ascertain its enduring influence on antimicrobial susceptibility patterns and infection-related mortality. Therefore, we recommend that all healthcare centers adopt ASP and its relevant interventions based on the most common practices in the healthcare setting, closely follow its impacts, and adjust interventions accordingly to combat antimicrobial resistance and preserve last-line therapeutic regimens.

## Data Availability

Due to privacy and ethical concerns, the data from our surveillance are not publicly available; however, anyone interested in using the data for scientific purposes can request permission from the corresponding authors (saedzyoud@yahoo.com; a.sabatin@najah.edu).

## Abbreviations

MDR: Multidrug-resistant
ASPs: Antibiotic stewardship programs
U.S: United States of America
IDSA: Infectious Diseases Society of America
SHEA: The Society for Healthcare Epidemiology of America
AMS: Antimicrobial Stewardship Program
AMR: Antimicrobial resistance
MDR-BSIs: Hospital-acquired multi-drug resistant-bloodstream infections
ICUs: Intensive care units
BMT: Bone marrow transplant
IRB: Institutional Review Board
SPSS: Statistical package for social sciences program
DOT: Days of therapy
NIS: New Israeli Shekel
S: Sensitive
R: Resistant
%: Sensitivity rate
ID: Infectious disease
USD: United States Dollar
MRSA: Methicillin-resistant *Staphylococcus S. aureus*
CRE: Carbapenem-resistant Enterobacteriaceae
